# Supplementary material for: Identification of photoperiod-induced specific miRNAs in the adrenal glands of Sunite sheep (Ovis aries)
Source: Front Vet Sci. 2022 Jul 22;9:888207. doi: 10.3389/fvets.2022.888207 (PMC9354845; doi:10.3389/fvets.2022.888207)
Supplement: Supplementary file 1 [file Data_Sheet_1.ZIP › Supplementary file/miRDeep2 file.zip!/miRDeep2 file/Know_Structure/SPLP42A1/oar-let-7a.pdf]

miRBase precursor : oar-let-7a
Total read count : 373762
oar-let-7a read count : 372294
remaining reads : 1468

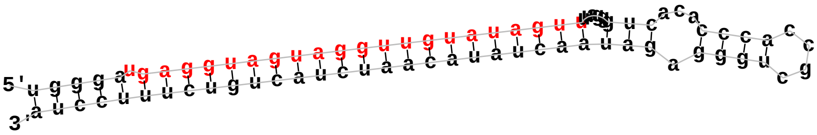

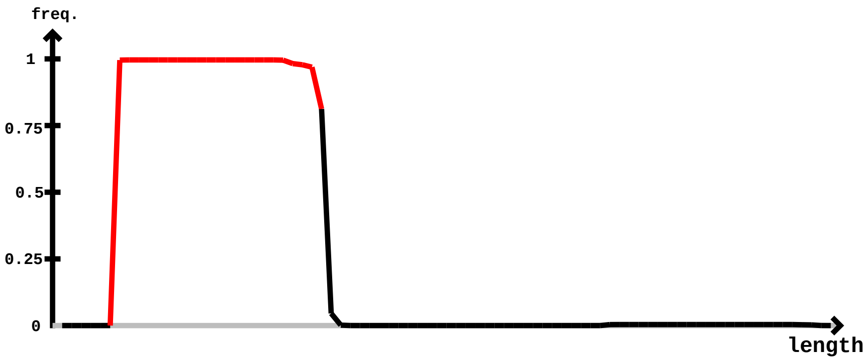

oar-let-7a

5'- ugggaugagguaguagguuguauaguuuuagggucacacccaccgcugggagauaacuauacaaucuacugucuuuccua -3'	exp		
(((((.(((((((((((((((((((((.....(((...((((....)))).)))))))))))))))))))))))))))))	reads	mm	sample
...gUugagguaguagguuguauaguu...	1	1	seq
....augagguaguagguuguau...	1	0	seq
....Uugagguaguagguuguauag...	1	1	seq
....Uugagguaguagguuguauagu..	4	1	seq
....augagguaguagguuguauagu..	3	0	seq
....augagguaguagguuguauaguu...	12	0	seq
....Uugagguaguagguuguauaguu...	4	1	seq
....Uugagguaguagguuguauaguuu..	1	1	seq
.....ugagguaguaAguugua..	10	1	seq
.....uUagguaguagguugua..	1	1	seq
.....ugagguaguagguugua..	281	0	seq
.....ugagguaguagguuUua..	1	1	seq
.....ugagguagGagguugua..	37	1	seq
.....ugaAguaguagguugua..	2	1	seq
.....ugagguagGagguuguau...	402	1	seq
.....ugagguaguagguuguau...	4342	0	seq
.....ugagguaguaAguuguau...	18	1	seq
.....ugagguaguagguuUuau...	13	1	seq
.....ugaAguaguagguuguau...	35	1	seq
.....ugagguUguagguuguau...	2	1	seq
.....ugagguagGagguuguaua..	177	1	seq
.....ugagguaguagguuguaua..	1451	0	seq
.....ugagguagGagguuguauag...	543	1	seq
.....ugagguaguagguuguauag...	2787	0	seq
.....ugagguagGagguuguauagu..	8089	1	seq
.....ugagguaguagguuguauagu..	50583	0	seq
.....ugagguagGagguuguauaguu...	28252	1	seq
.....ugagguaguagguuguauaguu...	258015	0	seq
.....ugagguaguagguuguauaguuu..	16420	0	seq
.....ugagguaguagguuguauaguuuu...	504	0	seq
.....ugagguaguagguuguauaguuuA...	81	1	seq
.....ugagguaguagguuguauaguuuua..	6	0	seq
......gagguaguagguuguau...	1	0	seq
......gagguagGagguuguauag...	1	1	seq
......gagguaguagguuguauag...	1	0	seq

oar-let-7a

```
ugggaugagguaguagguuguauaguuuuagggucacacccaccgcugggagauaacuauacaaucuacugucuuuccua
......gagguaguagguuguauagu......................................................   25    0    seq
......gagguagGagguuguauagu......................................................   5     1    seq
......gagguaguagguuguauaguu.....................................................   142   0    seq
......gagguagGagguuguauaguu.....................................................   25    1    seq
......gagguaguagguuguauaguuu....................................................   11    0    seq
......gagguaguagguuguauaguuuu...................................................   1     0    seq
.......agguaguagguuguauaguuu....................................................   1     0    seq
..........uaguagguuguauaguu.....................................................   1     0    seq
..........uaguagguuguauaguuuua..................................................   1     0    seq
...........................uuagggucacacccaccgcuggg..............................   1     0    seq
...........................uuagggucacacccaccgcuggga.............................   13    0    seq
...........................uuagggucacacccaccgcugggagaua.........................   1     0    seq
.....................................................uaacuauacaaucuacugucuuu....   2     0    seq
......................................................aacuauacaaucuacugucuuu....   3     0    seq
........................................................cuauacaaucuacuguc.......   1     0    seq
........................................................cuauacaaucuacugucu......   2     0    seq
........................................................cuauacaaucuacugucuu.....   4     0    seq
........................................................cuauacaaucuacugucuuu....   263   0    seq
........................................................cuauacaaucuacugucuuuc...   233   0    seq
........................................................cuauacaaucuacuguUuuuc...   1     1    seq
........................................................cuauacaaucuacugucuuucc..   860   0    seq
........................................................cuauacaaucuacugucuuuccu.   29    0    seq
.........................................................uauacaaucuacugucuuu....   1     0    seq
.........................................................uauacaaucuacugucuuuc...   3     0    seq
.........................................................uauacaaucuacugucuuucc..   12    0    seq
.........................................................uauacaaucuacugucuuuccu.   34    0    seq
..........................................................auacaaucuacugucuuucc..   1     0    seq
..........................................................auacaaucuacugucuuuccu.   3     0    seq
..........................................................auacaaucuacugucuuuccua   1     0    seq
```
